# Microplastics in the surface sediments from the eastern waters of Java Sea, Indonesia

**DOI:** 10.12688/f1000research.17103.1

**Published:** 2019-01-24

**Authors:** Defri Yona, Syarifah Hikmah Julinda Sari, Feni Iranawati, Syamsul Bachri, Wulan Cahya Ayuningtyas

**Affiliations:** 1 Marine Science Department, Faculty of Fisheries and Marine Science, Brawijaya University, Malang, Jawa Timur, 65145, Indonesia; 2 Marine Resources Exploration and Management (MEXMA) Research Group, Brawijaya University, Malang, Jawa Timur, 65145, Indonesia; 3 Geography Department, Faculty of Social Science, State University of Malang, Malang, Jawa Timur, 65145, Indonesia

**Keywords:** Anthropogenic activities, Java Sea, Microplastics, Surface Sediments

## Abstract

**Background:** This study aimed to investigate the abundance of microplastics in the eastern water of Java Sea. The study areas are well known for the high population and high industrial activities that can contribute to the plastic pollution.

**Methods:** Microplastics were sampled from the surface sediments at five different stations representing different local activities: fish landing area (St 1), mangrove forest (St 2), abandoned shrimp pond (St 3), river mouth (St 4) and open sea (St 5).

**Results:** Three types of microplastics were found; the most common was plastic fragments (54.34±6.39%) followed by fibers (41.45±4.59%) and films (4.21±3.90%). The highest abundance of microplastics was observed in the mangrove area (896.96±160.28 particles/kg), dominated with fragments and fibers. Films were found in greatest quantities in the fish landing area, but compared to the other types of microplastics, the abundance was much lower (80.73±37.62 particles/kg). Domestic wastes and fisheries activities were the main causes of the high microplastics in the study areas.

**Conclusions:** The results of this study showed that microplastic pollution is a serious problem that needs to be paid attention not only from the government but also from the local people. Plastics management waste is needed.

## Introduction

The presence of microplastics in the aquatic environment has become a global concern. Microplastics are small pieces of plastic less than 5 mm in size ( Barnes *et al*., 2009; Hidalgo-Ruz *et al*., 2012). There are two types of microplastics based on their source, the primary microplastics from manufactured plastics in microscopic sizes such as scrubbers and pellets ( Isobe, 2016), or secondary microplastics which derived from the breakdown of bigger plastic products such as fragments, fibers or films ( Zhu *et al*., 2018; Zobkov & Esiukova, 2017).

Once in the aquatic environment, microplastics might float in the water column or sink to the bottom, depending on the particle density ( Barnes *et al*., 2009; Kowalski *et al*., 2016). There have been many studies conducted to analyze the presence of microplastics in the water ( Chae *et al*., 2015; Isobe *et al*., 2015; Iwasaki *et al*., 2017), sediments ( Alomar *et al*., 2016; Imhof *et al*., 2017; Wang *et al*., 2017; Zobkov & Esiukova, 2017) or both ( Frère *et al*., 2017; Zhu *et al*., 2018). The sinking behavior of microplastics to the bottom sediments might be the result of biofouling, which can increase its density, the size and shape of microplastics and also fluid density ( Kowalski *et al*., 2016).

Sediments have been considered to be major sinks of microplastics. With the capability of microplastics to concentrate other organic pollutants or heavy metals and also their durability and resistance to degradation, the accumulation of microplastics in sediments can bring harm to marine and human life. Thus, this study was conducted to evaluate microplastics contamination in the eastern water of Java Sea, which is busy with many human activities. The types of microplastics and the abundances were used to investigate the influence of anthropogenic factors on the spatial distribution of microplastics in the study areas.

## Methods

### Sampling region

**Figure 1.**
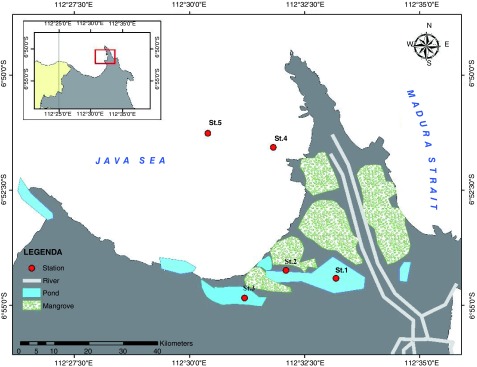
Sampling location of microplastics in the surface sediments from the eastern waters of the Java Sea.

Sampling was conducted in the eastern water of Java Sea, Gresik, Indonesia. There were five sampling stations that represented different local activities. Station 1 was located in the fish landing area that is busy with fisheries activity, especially in the morning. Station 2 was located in the mangrove area that is frequently inundated during high tide. The mangrove area is vulnerable to the plastic wastes from other places that are carried away by the current and tides. Station 3 was located in the abandoned shrimp pond and was once known for shrimp culture. However, this activity had stopped and many ponds have been abandoned. Some local people discard their waste in these ponds, including plastic waste. Station 4 was located in the river mouth of Bengawan Solo River that connected to the open sea. Bengawan Solo River is the longest river in the Java Island, passing through many cities in the Central and East Java Provinces and along the way could bring domestic wastes to its end point in the Java Sea. Station 5 was located in the open sea, about 1 km from the river mouth ( Figure 1).

### Sampling method

Sampling was conducted in March 2018 and samples were collected in the surface sediment using an Ekman Grab soft sediment sampler. In total, three replicates were obtained and about 500 g of sediment samples from each replicate of the sampling station were stored in sealed plastic bags. Sediment samples were taken to the laboratory for further analysis. For more information about the study areas regarding the present of the plastic wastes, we also held informal discussions with the local fishermen, especially those who have concern for the mangrove ecosystem. Since this was considered low-risk personal communication, ethical approval and consent were not sought.

### Microplastic analysis

Microplastics analysis was conducted by modifying the section 3.5 of the NOAA method ( Masura *et al*., 2015). In the laboratory, 150 g of sediment samples were oven-dried for 24 hours at 90°C. Density separation was performed by adding 20 ml 0.05 M Fe(II) and 20 ml 30% H _2_O _2_. The samples were then homogenized on a hotplate with stirrer for 30 minutes at 60°C. To remove organic materials, an additional 20 ml of 30% H _2_O _2_ and NaCl were added and left overnight. After one night, the floating microplastics were collected by filtration using an 0.3-mm mesh filter. Visual identification of microplastics was conducted under a microscope with three distinct rules to separate the types of microplastics. A fragment is a particle that cannot be torn apart with tweezers, with sharp and broken edges of irregular shape and size of degradational plastic; a fiber is a particle that equally thick throughout the entire length and is not tapered at the end; and film that is very thin, part of the sheets of plastic bags and similar ( Dai *et al*., 2018; Hidalgo-Ruz *et al*., 2012; Nor & Obbard, 2014; Zobkov & Esiukova, 2017). The results of this study were compared with the results from other studies to understand more of the sources of plastic pollution.

### Statistical analysis

Normality test was performed to determine the data distribution and to decide whether to use parametric or nonparametric tests for the statistical analysis. The abundance of microplastics proven to be distributed normally, thus, one-way ANOVA was used to compare the abundance of microplastics among the sampling stations (p < 0.01) and the post hoc Tukey`s test was run to confirm the differences of microplastics between sampling stations. Kruskal-Wallis H test was conducted to test the difference in type of microplastics found in the study areas in which the distributions were found to be nonparametric. All statistical tests were carried out using SPSS 16.0 for Windows.

## Results and discussion

### Location of microplastic detection

**Figure 2.**
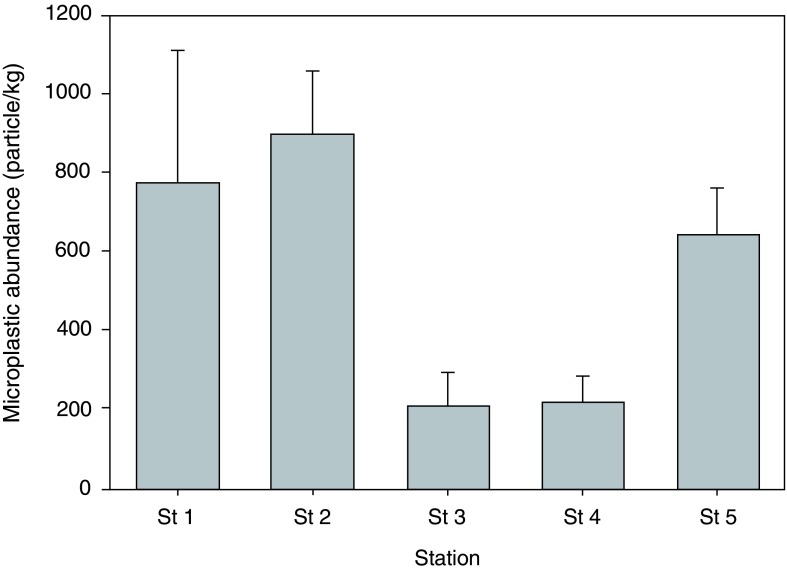
The abundance of microplastics in each sampling site (n=3).

Microplastics were detected in the surface sediments of the sampling stations in the eastern waters of Java Sea. Anthropogenic activities mostly contributed to the present of microplastics in the study areas. The areas have been known to be highly populated. This is also home to many industrial activities which discharge wastewater to the eastern water of the Java Sea. Microplastics appeared in the range of 206.04−896.96 particle/kg ( Figure 2). The highest abundance of microplastics was found at Station 2 in the mangrove area (896.96±160.28 particles/kg), followed by Station 1 (772±336.75 particles/kg) and Station 5 (639.51±121.58 particles/kg). Stations 3 and 4 were observed to contain rather similar amounts of microplastics, which were three times lower than the abundance in the mangrove area (206.04±84.49 particles/kg and 215.54±64.58 particles/kg, respectively). According to one-way ANOVA, there was a statistically significant different in the abundance of microplastics among sampling stations (p < 0.01). Tukey’s post-hoc test revealed that the abundances of microplastics at Stations 1 and 2 were significantly different to the abundances at Stations 3 and 4 (p = 0.05). There was no statistically significant difference between the abundance of microplastics at Station 5 and the other stations. Raw data on microplastic abundance, along with all other raw data, are available on OSF ( Yona, 2018).

Microplastics in the mangrove ecosystems have been studied in the Singapore`s coastal areas ( Nor & Obbard, 2014), where there were much lower amounts compared to those found in this study. Even though the abundances were very much different between these studies, but the reasons of the occurrence of the microplastics in the mangrove ecosystems were rather similar. High occurrence of microplastics at Station 2 in the mangrove area was observed, which could be the result of root system of mangroves, which can trap many different type of rubbish including plastics. Moreover, tides that frequently inundated mangrove beds could bring more plastic wastes from the surrounding waters.

Due to their small size and ability to float in the water column, microplastics can be transported for long distance by ocean currents ( Iwasaki *et al*., 2017). Eastern water of Java Sea is the end point of a very big river, Bengawan Solo, which passes many cities in Java Island and carried plastic wastes along its way to the ocean. Therefore, microplastics found in this study might not just from the local sources, but also from faraway places. According to information from local fishermen, during west monsoon season (November-February), mangroves in the study area are filled with plastic waste from the Bengawan Solo River. Similar results were also found in the study in the Saigon River canal system crossing a megacity, Ho Chi Minh City, in which the source of the plastic pollution was from the land-based due to local habits and waste management ( Lahens *et al*., 2018).

On the other hand, limited interaction of Station 3, which located in the shrimp pond, with the surrounding waters resulted in a low abundance of microplastics. Even so, the abundance of microplastics in the pond was not that low (206.04 particles/kg). This may be because the pond has been abandoned for quite some times and some people from local village may have discarded their rubbish inside the pond.

### Types of microplastics found

**Figure 3.**
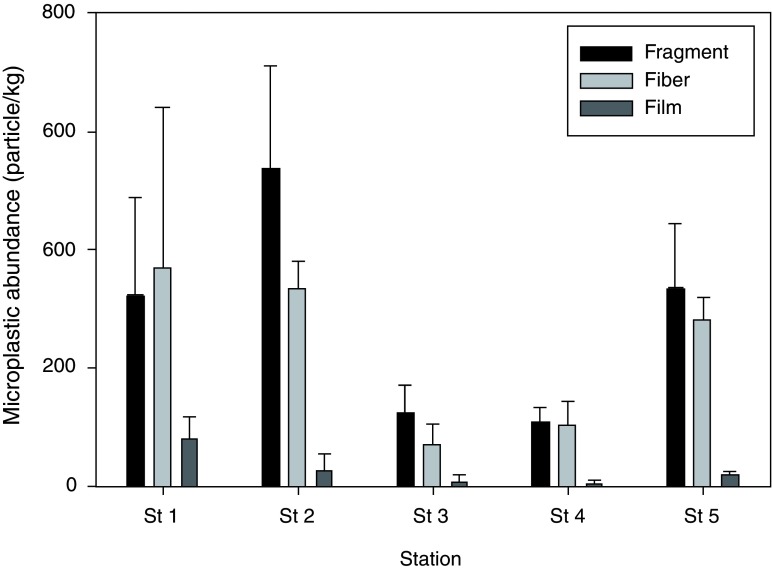
The average abundance (n=3) of the type of microplastics collected from the eastern water of Java Sea.

There were three type of microplastics found in the study areas: plastic fragments, plastic fibers and plastic films ( Figure 3). Fragments and fibers dominated most of the sampling stations, while films occurred in very low number compared to the other two types of the microplastics. In total, half of the microplastics found were plastic fragments (54.34±6.39%), followed by plastic fibers (41.45±4.59%) and plastic films (4.21±3.90%). Kruskal-Wallis H test showed that there was a statistically difference in the type of microplastics (fragment, fiber and film) among the study sites (p < 0.01).

Fragments contributed the most to the composition of microplastics in the study areas. This type of microplastic is the result of fragmentation of large plastic pieces into smaller particles ( Alomar *et al*., 2016), and mostly the contribution comes from the domestic waste. Fisheries activity in the study areas might contribute to the present of fiber, as mostly local fishermen use plastic fishing nets to catch their fish. A high abundance of fibers was also observed in the North Yellow Sea as the result of constant use of plastic fishing and nets and ropes as the main fishing tools ( Zhu *et al*., 2018).

The highest abundance of plastic fragments appeared at Station 2 in the mangrove area (537.25 ± 160.28 particle/kg). The highest abundance of plastic fibers was also found at Station 2 compared to the other stations. On the other hand, plastic films were detected the highest at Station 1 in the fish landing area.

### Comparison with the results of other studies

Compared to the results from the other studies ( Table 1), the levels of microplastics found in this study were similar to values obtained in the Western Mediterranean Sea ( Alomar *et al*., 2016) and much higher than those in the North Yellow Sea ( Zhu *et al*., 2018). The dominant type of microplastics found among the studies was also different. The study in the North Yellow Sea found that plastic films predominated; in the Bohai Sea the most common type was plastic fibers ( Dai *et al*., 2018), while this study obtained the highest percentage of plastic fragments. The results revealed that microplastics found in this study are mostly from the degradation of the plastic wastes from human activities as stated by Barnes *et al*. (2009) that fragments are the result of the breakdown of a wide range of everyday plastic products. The lack of awareness from the citizen on how dangerous plastic materials are to the environment is the main reason for plastic pollution ( Derraik, 2002). Efforts from the government and the community are therefore needed to combat plastic use and production.

**Table 1.** The abundance of microplastics in the sediment reported from this and other studies.

Location	Total abundance	Percentage (%)			Reference
		Fragment	Fiber	Film	
Beijiang River	178–544 items/kg	–	–	–	Wang *et al*. (2017)
The North Yellow Sea	37.1±42.7 items/kg	–	39.1±22.3	58.1±24.9	Zhu *et al*. (2018)
Western Mediterranean Sea	100.78–897.35 particle/kg	–	–	–	Alomar *et al*. (2016)
Bohai Sea, China	31.1–256.3 pieces/kg	9.0	83.7	1.1	Dai *et al* (2018)
Bohai and Yellow Sea	72–171.8 items/kg	2.55	93.88	1.53	Zhao *et al*. (2018)
Eastern water of Java Sea	206.04–896.96 particle/kg	54.34±6.39	41.45±4.59	4.21±3.90	**This study**

## Conclusion

Microplastics were found in all of the samples from the study area, with the highest levels found in the mangrove area. Fragments were the most common type of microplastic observed, followed by fibers and then small amount of films. The results showed that plastic contamination in the eastern waters of the Java Sea were mostly from anthropogenic activities, especially domestic waste. This plastic waste was not just from the local sources but also from the long-distance sources carried away by the Bengawan Solo River that end in the eastern water of Java Sea.

## Data availability

Raw data on the microplastics at each location are given on OSF. DOI: https://doi.org/10.17605/OSF.IO/H3ZDQ ( Yona, 2018).

Data are available under the terms of the Creative Commons Zero "No rights reserved" data waiver (CC0 1.0 Public domain dedication).
