# Supplementary material for: Microplastics in the surface sediments from the eastern waters of Java Sea, Indonesia
Source: F1000Res. 2019 Jan 24;8:98. [Version 1] doi: 10.12688/f1000research.17103.1 (PMC6426082; doi:10.12688/f1000research.17103.1)
Supplement: Peer review history

# Peer review: Reviewer response for version 1

**DOI:** 10.5256/f1000research.18701.r43573

**Published:** 2019-03-19

**Authors:** Alex Sen Gupta

**Affiliations:** 1 Climate Change Research Centre (CCRC), ARC Centre of Excellence for Climate System Science, University of New South Wales (UNSW), Sydney, New South Wales, Australia

Microplastics in the surface sediments from the eastern waters of Java Sea, Indonesia

 Defri Yona, Syarifah Hikmah Julinda Sari, Feni Iranawati, Syamsul Bachri, Wulan Cahya Ayuningtyas

 This paper uses coastal sediment samples from sites in the east Java Sea to examine abundance and composition of microplastics. The article is generally well written although I note below a few places where the language could be improved. The work really constitutes a report rather than a scientific article as while it provides useful baseline information, it does not provide any mechanistic advance, or specific policy advice. I would suggest this article is sound subject to minor revisions.

**Abstract:**

- “that needs to be paid attention” > “that needs greater attention”.
- “Plastics management waste is needed.” > “Plastics waste management is needed.”

 

**Page 3:**

 Paragraph 1:

- “the primary microplastics from manufactured plastics”. All plastics are manufactured, do you mean “used in the manufacture of plastic products"? Suggested edit: "There are two types of microplastic: primary microplastics including pellets and scrubbers that are used in the manufacture of products and secondary microplastics which are...".

 

 Paragraph 2:

- “and also fluid density” > “and also by fluid density”.

 

 Paragraph 3:

- “Sediments have been considered to be major sinks of microplastics.” Reference needed.
- “With the capability of microplastics to concentrate other organic pollutants or heavy metals and also their durability and resistance to degradation, the accumulation of microplastics in sediments can bring harm to marine and human life.” Reference needed or rephrase e.g. “could potentially cause harm...”.
- “Thus, this study was conducted to evaluate microplastics contamination in the eastern water of Java Sea, which is busy with many human…” > “Thus, this study was conducted to evaluate microplastics contamination in the eastern water of the Java Sea, which is subject to intensive human…”.

 

 Paragraph 4:

- “that are carried away by the current and tides” > “that are carried by the currents and tides”.
- “shrimp pond and was once” > “shrimp pond that was once”.
- “this activity had stopped” > “this activity has stopped”.
- “in the Central and East Java Provinces and along the way could bring domestic wastes to its end point in the Java Sea.” > “in Central and East Java Provinces and along its path could accumulate domestic wastes that ultimately is transported to the Java Sea.”
- It would be useful to give a brief introduction to the local oceanography, as this would help to understand the distribution of microplastics: what does the circulation look like? How important are tides and tidal currents?

 

 Paragraph 5:

- “In total, three replicates were obtained and about 500 g of sediment samples from each replicate of the sampling station were stored” > “In total, three replicates were obtained at each site comprising of about 500 g of sediment per replicate. Samples were stored…”.
- “the present of the plastic wastes” > “the presence of plastic waste…”.

 

 Figure 1:

- I’ve looked at a number of other maps of this region (to try and orient myself) and the coastline looks quite different to the one shown here (e.g. see google map satellite). The inset map isn’t very helpful, the red square doesn’t have the correct size. It would be also be more helpful to zoom out to show east Java and Madura island. What is the yellow area in the inset map?

 

**Page 4:**

 Paragraph 4:

- “Anthropogenic activities mostly contributed to…”. But aren’t all plastics related to anthropogenic activity?
- “activities mostly contributed to the present of” > “activities mostly contributed to the presence of”.
- “The areas have been known to” > “The areas are to”.
- “206.04−896.96 particle/kg”. You don’t need so many significant figures. The nearest gramme would be more than adequate given the huge range. Can you explain the units? Is this no. of particles per kg of water? I know this has been commonly used but I’m not clear as to the relevance of using particles as a measure. Are 10 small particles considered the same as 10 large particles?
- “mangrove area (896.96Å}160.28 particles/kg)”. Above the upper limit for the range was 896.96. Here you have 896+/-160 so the total range is higher than stated above. Needs to be clearer what you mean by ‘range’.
-  “three times lower”. 200x3=600. Its more than 4 times.

Figure 2: what are the error bars? The total range? Presumably they should go in both directions. Makes more sense to show the actual values of the 3 replicates.

**Page 5:**

 Paragraph 1:

- “in the Singapore`s coastal areas” > “in Singapore`s coastal areas”.
- “these studies, but the reasons” > “these studies, the reasons”.
- “Even though the abundances were very much different between these studies, but the reasons of the occurrence of the microplastics in the mangrove ecosystems were rather similar.”
- How do you know this? Either you have evidence for this or it is just a guess, in which case you need to use more careful language, e.g. “In both cases we suspect that the high abundance relates to large tidally driven transport of water through the mangroves and the capture of particles by the complex root systems.” NB you haven’t provided any information about the tidal circulation.

 

 Paragraph 2:

- “…can be transported for long distance by ocean currents (Iwasaki *et al.*, 2017). Eastern water of Java Sea is the end point of a very big river, Bengawan Solo…” I don’t follow the link. In the first sentence you are talking about ocean currents. In the second you are referring to estuarine flow.
- “and carried plastic wastes” > “and carries plastic wastes”.
- “might not just from” > “might not just come from”.
- “but also from faraway places” > “but may have been transported considerable distances”.
- “during west monsoon” > “during the west monsoon”.
- “Similar results were also found in the study in the Saigon River canal” > “The importance of estuarine contamination was also found in a study of the Saigon River canal”.
- “pollution was from the land-based” > “pollution was land-based”.

 

 Paragraph 3:

- “which located” > “which is located”.
- “resulted in a low abundance of microplastics” > “resulted in a low abundance of microplastics relative to the other sites.”
- “and some people from local village may have discarded their rubbish inside the pond.” > “and dumping of waste from the local village into the pond may have occurred.”

 

 Paragraph 4:

- “while films occurred in very low number compared to the other two types of the microplastics.” > “while films occurred in very low numbers in comparison.”

 

 Paragraph 5:

- “Fragments contributed the most to the composition of microplastics in the study areas”. You just said this, no need to repeat. 
- “and mostly the contribution comes from the domestic waste.” > “with the largest contribution coming from domestic waste.”
- “to the present of fiber, as mostly local fishermen use” > “to the presence of fiber, as local fishermen primarily use”.
- “fishing and nets” > “fishing nets”.

 

 Paragraph 6:

- “were detected the highest at Station 1” > “were most common at Station 1”.

**Page 6:**

- Table 1: What’s the difference between an item and a particle?
- “…North Yellow Sea … in the Bohai Sea…”. Describe where these are and the local setting.
- Fibres seem to be overwhelming in Bohai - did the authors explain what the source was? Would be nice to mention here. Also what are the source of films in the north Yellow Sea? Would be interesting to offer some explanation as to why the sources are different in different areas.

 

 Paragraph 1:

- “The lack of awareness from the citizen on how dangerous plastic materials are…”. So what are the dangers? 

Paragraph 2:

- “…mostly from anthropogenic activities…” What plastic isn’t related to anthropogenic activity?
- “carried away by” > “carried by”.

There seems to be a lot of speculation. Why does station 4 have so much less plastic than station 5, if the primary source is from the estuary?

I have read this submission. I believe that I have an appropriate level of expertise to confirm that it is of an acceptable scientific standard.

# Peer review: Reviewer response for version 1

**DOI:** 10.5256/f1000research.18701.r43569

**Published:** 2019-02-06

**Authors:** Atsuhiko Isobe

**Affiliations:** 1 Research Institute for Applied Mechanics (RIAM), Kyushu University, Kasuga, Japan

This is a well-written paper on microplastic abundance in the bottom sediment east of the Java Sea. I have only trivial points to be improved. 

- P. 3, "Methods": Please add "Bengawan Solo River" into Figure 1. 
- P. 4, "Microplastic analysis": What was the maximal size of microplastics in your analysis? I recognized that the minimal sizes was 0.3 mm according to the mesh filter. The maximal size was however not specified.
- P.4, "Microplastic analysis": How did you avoid the contamination of fibers floating in the air in both the field and laboratory? We sometimes set a "blank" to monitor air-borne microfibers during the experiment. If you did not conduct the monitoring, please add the sentence: the abundance of microfibers detected in this study might be overestimated by the contamination due to air-born microfibers.    

I have read this submission. I believe that I have an appropriate level of expertise to confirm that it is of an acceptable scientific standard.

# Response

**Published:** 2019-02-11

**Authors:** Syarifah Sari

**Affiliations:** Brawijaya University, Indonesia

We would like to say thank you to Prof. Atsuhiko Isobe for the comments and suggestions. Our responses to his concerns on making the improvements to our paper are presented below:

- P. 3, "Methods": Please add "Bengawan Solo River" into Figure 1. 
  - We will add the Bengawan Solo River into Figure 1 in our next revised version of the manuscript. 
- P. 4, "Microplastic analysis": What was the maximal size of microplastics in your analysis? I recognized that the minimal sizes was 0.3 mm according to the mesh filter. The maximal size was however not specified
  - The maximal size was 5 mm. We conducted filtration using stacked arrangement of sieves in the size of 5 mm and 0.3 mm to collect microplastic samples.
- P.4, "Microplastic analysis": How did you avoid the contamination of fibers floating in the air in both the field and laboratory? We sometimes set a "blank" to monitor air-borne microfibers during the experiment. If you did not conduct the monitoring, please add the sentence: the abundance of microfibers detected in this study might be overestimated by the contamination due to air-born microfibers.
  - We did not conduct the monitoring of air-born microfibers, therefore, we will add your suggestion to the revised manuscript.
